# Cardiac Catheterization for Coronary Artery Fistulas in Children: Evaluation, Management, and Outcomes—A Single-Center Experience

**DOI:** 10.3390/jcdd13020091

**Published:** 2026-02-12

**Authors:** Hayrettin Hakan Aykan, Nilay Korgal, Alpay Çeliker, Tevfik Karagöz

**Affiliations:** 1 Division of Pediatric Cardiology, Department of Pediatrics, Faculty of Medicine, Hacettepe University, Ankara 06100, Türkiye; 2 Life Support Center, Hacettepe University, Ankara 06100, Türkiye; 3 Department of Pediatric Cardiology, VKV American Hospital, İstanbul 34365, Türkiye

**Keywords:** coronary artery fistula, pediatric cardiology, transcatheter closure, congenital heart disease, cardiac catheterization

## Abstract

Coronary artery fistulas (CAFs) are rare congenital coronary anomalies in children and are frequently detected incidentally; however, the optimal management of asymptomatic cases and long-term outcomes remain debated. We retrospectively evaluated patients <18 years who underwent cardiac catheterization and coronary angiography for CAF at a single tertiary center between 2003 and 2022, analyzing demographic, clinical, angiographic, procedural, and follow-up data; fistulas were classified using a modified Sakakibara system, and temporal changes in institutional clinical approach and device selection were also assessed. Forty-two patients (mean age 7.4 ± 5.9 years) were included, most of whom were asymptomatic (80.9%); the left coronary artery was the most frequent origin and 85% drained to right-sided chambers. Transcatheter closure was attempted in 25 patients and was technically successful in 23 (92%); complete occlusion was achieved angiographically in 61% immediately and exceeded 90% during follow-up due to spontaneous resolution of residual shunts. One patient required surgery for persistent moderate residual flow, and no major procedural complications, thrombotic events, or ischemic outcomes were observed. In selected children, transcatheter CAF closure is safe and effective, while conservative follow-up appears appropriate for small, hemodynamically insignificant fistulas, supporting individualized, anatomy-guided management.

## 1. Introduction

Coronary artery fistulas represent an uncommon congenital anomaly, particularly in the pediatric population, where one or more coronary arteries abnormally communicate with a cardiac chamber, great vessel, or other vascular structure, bypassing the myocardial capillary bed. These fistulous connections can arise from any of the major epicardial coronary arteries and drain into various locations (heart chambers, pulmonary artery, vena cava, etc.).

Coronary angiography studies have reported that the prevalence of coronary artery fistulas ranges from 0.13% to 0.6%, but the estimated incidence in the general population is approximately 0.002% [1,2,3]. In patients with congenital heart disease, the prevalence of coronary artery fistulas is reported to be around 0.08–0.4% [4,5,6,7]. The true incidence of coronary artery fistulas in children is difficult to determine, as the majority of cases in the pediatric age group are typically asymptomatic, and the need for coronary angiography is rare in this patient population. However, advancements in transthoracic echocardiographic imaging have enabled the diagnosis of many pediatric patients with coronary artery fistulas, even when the fistula is small, through routine echocardiographic examinations performed independent of fistula-related symptoms. Echocardiography-based studies have also revealed the rarity of coronary artery fistulas in the pediatric population, with reported prevalence rates ranging from 0.01% to 0.06% [7,8,9].

Coronary artery fistulas can be classified according to their anatomical characteristics, including their origin from a specific coronary artery, the location of their drainage site (e.g., right atrium, right ventricle, pulmonary artery), the size and tortuosity of the fistulous connection, and the presence of any associated cardiac anomalies. While the clinical presentation of coronary artery fistulas can vary significantly based on factors such as patient age, fistula size, and the specific anatomy of the anomalous connection, the majority of small coronary artery fistulas tend to remain asymptomatic, particularly during the first two decades of life [10]. However, larger fistulas may manifest with a range of symptoms, including exertional dyspnea, fatigue, chest pain, and, in some cases, even congestive heart failure. Furthermore, the abnormal flow dynamics within the fistulous connection can predispose to complications such as thrombosis, myocardial infarction, aneurysm formation, rupture, endocarditis or death [9,10,11].

Traditionally, surgical ligation or division of the fistula was the standard treatment approach, requiring open-heart surgery with cardiopulmonary bypass, which carries inherent risks and potential morbidities. However, with advancements in interventional cardiology techniques and device technology, transcatheter closure has emerged as a safe and effective alternative to surgical repair for many coronary arteriovenous fistulas in pediatric patients [12,13,14]. Transcatheter closure offers several advantages over surgery, including avoidance of a thoracotomy, reduced hospital stay, and decreased recovery time, thus minimizing the psychological impact on young patients and their families.

Over time, evolving closure approaches and follow-up data have indicated that currently, small, asymptomatic (without chest pain, ventricular dysfunction, exercise intolerance or respiratory symptoms) fistulas that do not lead to cardiac chamber dilation should be monitored, as these have a low probability of expansion and some may even close spontaneously [8,9,15]. However, there is ongoing discussion that even asymptomatic moderate-sized fistulas, and rarely small fistulas as well, may become problematic in later stages of life, and the procedures performed at that time may carry higher risks. Accordingly, the decision to intervene should be predicated on a thorough evaluation of the risks and benefits specific to each individual patient.

The aim of this study was to retrospectively evaluate patients under 18 years of age who underwent cardiac catheterization with the diagnosis of coronary artery fistula in our center, to interpret the characteristics of fistulas in these patients according to current classifications, and to discuss the management of this patient group based on the follow-up data of patients who underwent transcatheter closure and were managed conservatively.

## 2. Materials and Method

### 2.1. Study Design

In this study, we retrospectively evaluated pediatric patients (<18 years) who underwent diagnostic or interventional cardiac catheterization with coronary angiography for coronary artery fistulas at our center between 2003 and 2022. Forty-two patients whose diagnosis was confirmed by coronary angiography were included in the study. Demographic, clinical, and procedural data were obtained from both paper-based and electronic medical records. All available angiographic images and reports were reanalyzed to reassess fistula origin, course, and drainage site. For the 6 patients without available image records, evaluations were based on written reports. The study protocol was approved by the Ethics Committee of Hacettepe University Faculty of Medicine (Research No: SBA 25/416, Approval No: 2025/10-22, Approval Date: 6 May 2025).

### 2.2. Fistula Classification

Fistulas were classified according to a modified Sakakibara classification system [16]. Type A fistulas originated from the proximal third of the native vessel, while Type B fistulas arose distally beyond the proximal third or as a continuation of the native vessel. Fistulas originating from a single coronary branch, following a single course and draining into one region, were classified as simple, while fistulas with multiple origins and/or termination including plexiform variants were classified as complex fistulas. Fistulas were classified based on the diameter of the distal reference coronary artery. Those with a diameter less than 1 time the reference size were considered small, those between 1 and 2 times the reference were moderate, and those greater than 2 times the reference were classified as large [17]. The diameters of complex fistulas, characterized by multiple drainage sites, were not assessed using the size classification system described.

### 2.3. Procedure and Management

All procedures were performed under general anesthesia or deep sedation with local anesthesia, after obtaining informed consent from the patient’s legal representatives. Percutaneous femoral venous and arterial access was obtained in all patients. Following femoral arterial sheath placement, unfractionated heparin was administered at a dose of 100 units/kg. Activated clotting times were monitored and maintained between 200 and 300 s, with additional heparin administered as needed.

Following hemodynamic and oximetric studies, aortic root and selective coronary angiography were performed to delineate the anatomy of the fistula, in terms of the origin, course, size, drainage site and number of feeding vessels, as well as to identify any concomitant coronary anomalies. Immediate total occlusion was defined as the absence of flow beyond the fistula on angiographic assessment during the procedure or on echocardiography performed the day after. Residual shunts were assessed retrospectively based on transthoracic echocardiography reports using color Doppler imaging. Residual flow was evaluated subjectively and classified qualitatively as trivial or moderate.

For patients considered suitable for transcatheter closure (25 patient), the technical approach (retrograde-arterial, antegrade-venous, or arteriovenous loop) was selected based on the specific anatomy of the fistula. Device selection was influenced by the anatomy and diameter of the fistula, the proximity to normal coronary artery branches, as well as the available device options during that period. The occlusion devices used included various microcoils, coils (PFM Medical, Cologne, Germany; Cook Gianturco patent ductus arteriosus coil, Cook Cardiology, Bloomington, IN, USA), Amplatzer duct occluders (II, additional sizes and Piccolo), and Amplatzer vascular plugs (St Jude/AGA Medical, Golden Valley, MN, USA). *N*-butyl cyanoacrylate (NBCA) was used for occlusion in one patient. After deployment, contrast imaging was performed to detect any residual shunt and ensure native coronary artery patency.

### 2.4. Post-Procedure Care and Follow-Up

Following the procedure, all patients were hospitalized and monitored at least for 24–48 h. A 12-lead electrocardiogram (ECG) and transthoracic echocardiography were performed the day after device implantation. Aspirin therapy was initiated in all patients and continued for a minimum of six months. In two patients with distal fistula, aneurysmal dilatation of the coronary arteries, and slow flow or a blind pouch appearance within the coronary artery after device implantation, warfarin therapy was administered for 4–6 months along with aspirin. Aspirin treatment was continued in these patients based on the presence of coronary artery aneurysm. Scheduled follow-up visits were conducted at 1 month, 6 months, and 1 year post-procedure. According to the patients’ clinical conditions and needs, patients with persistent residual shunt were especially called for more frequent check-ups. If no complications were observed, subsequent follow-up intervals were tailored to the individual patient and ranged between 6 and 12 months. During each follow-up visit, patients underwent ECG and echocardiographic assessments. In cases where clinical suspicion warranted further investigation, additional imaging with coronary computed tomography angiography (CTA) or repeat catheterization was performed. The heterogeneity of follow-up imaging modalities reflects era-dependent practice patterns over the 20-year study period, as well as individualized clinical judgment and clinician preference based on patient age, clinical condition, procedural findings, and institutional experience.

### 2.5. Statistical Analyses

Statistical analyses were performed using IBM SPSS Statistics version 26.0 (IBM Corp., Armonk, NY, USA). For categorical variables, descriptive statistics were presented as frequencies and percentages. A One-Sample Kolmogorov–Smirnov Test was used to determine the normal distribution of the variables. Normally distributed continuous variables were presented as mean ± standard deviation (minimum–maximum), while non-normally distributed continuous variables were presented as median (minimum–maximum).

## 3. Results

### 3.1. Patient Characteristics

Between 2003 and 2022, a total of 42 patients diagnosed with coronary artery fistulas underwent diagnostic or interventional cardiac catheterization at our institution. The mean age at the time of catheterisaton was 7.4 ± 5.9 year (range: 3 days–18 years), with a median weight of 23 kg (range: 3.5–75 kg). The study group included 26 male and 16 female patients. The majority of patients (80.9%) were asymptomatic, while a notable proportion (76.1%) presented with a murmur. Other clinical findings encompassed shortness of breath, palpitation, and non-specific chest pain. One patient was diagnosed during evaluation for prolonged fever and suspected endocarditis. No patients exhibited ischemic ECG findings, while non-specific ST-T wave changes, not directly associated with the fistula, were observed in 3 patients. The general demographic and clinical characteristics of the patients are summarized in Table 1.

### 3.2. Fistula Characteristics

Angiographic evaluations were utilized to identify the origin and drainage locations of the fistulas. Our findings indicated that the majority of fistulas originated from the left coronary artery (71.4%), with the remaining arising from the right coronary artery. Of all fistulas, 36 (85%) drained into the right heart chambers. In only 6 (15%) cases, the fistulas drained into left heart chambers such as the left atrium or left ventricle, all of which originated from the left coronary artery. According to the Sakakibara classification, proximal (type A) fistulas were more common (54.7%), regardless of whether the fistulas originated from the right or left coronary artery. Among the 30 patients with simple morphology, the fistula size was classified as small in 12 patients, medium in 12, and large in 6. In 12 patients (28.6%), the coronary artery fistula had a complex morphology and was not subjected to diameter evaluation. The anatomic and morphologic characteristics of the coronary artery fistulas are summarized in Table 2.

### 3.3. Transcatheter Closure Outcomes

Following baseline angiographic evaluation, 25 patients underwent transcatheter fistula closure. Conversely, transcatheter closure was not performed in 17 patients for various reasons. Among these 17 patients, the decision not to proceed with intervention was primarily due to the hemodynamic insignificance of the fistula and the asymptomatic status of 14 patients, 12 of whom (85.7%) had coronary fistulas originating from the left coronary artery. In 2 patients, transcatheter intervention was deemed unsuitable because of the complex anatomy of the fistula and the potential risk of occluding normal coronary artery branches with the device. One of these two patients, presenting with a distally located fistula and a significant shunt, was subsequently referred for surgical intervention. Conversely, a decision was made to manage the other patient with non-interventional clinical monitoring, taking into account the magnitude of the shunt. A third patient necessitated surgical intervention due to concomitant aortic stenosis that was unresponsive to balloon valvuloplasty and was therefore referred for surgical management.

The median age and weight of the 25 patients who underwent transcatheter intervention was 4 years (3 days–18 years) and 23.7 kg (3.5–75), respectively. The mean pulmonary/systemic flow ratio was 1.6 ± 0.6 (1.2–2.9). In one case, a patient with a history of surgical correction for a significant coronary fistula in infancy required subsequent intervention due to the detection of fistula recanalization during follow-up (patient 4). Patient 7 received a diagnosis of infective endocarditis upon presentation and underwent fistula closure following a course of appropriate antibiotic therapy. Table 3 provides a detailed summary of the baseline characteristics, procedural information, and follow-up outcomes for the 25 patients who underwent transcatheter closure.

During transcatheter closure attempts, various devices were employed, including microcoils (*n* = 8), Amplatzer Vascular Plugs (*n* = 7), Amplatzer Duct Occluders (*n* = 6), NBCA (*n* = 1), coils (*n* = 1), and a combination of microcoils and coils (*n* = 1). The initial closure attempt involved the use of NBCA in 2003, which was subsequently followed by the implementation of microcoils, vascular plugs, and duct occluders in subsequent years. Depending on the anatomical characteristics of the fistula and the device to be used, a retrograde arterial approach was utilized for implantation in 19 of 25 patients, while an antegrade approach was preferred in 6 patients via the creation of an arteriovenous (AV) loop. In 3 of these patients, the alternative approach was chosen after the initial attempted approach proved unsuccessful.

Device implantation was technically successful in 23 (92%) cases, while the procedure was deemed unsuccessful in 2 patients. In the first of these patients (patient 6), failure was attributed to the inability to achieve catheter stabilization in the target region, leading to a decision for clinical monitoring. The other patient (patient 12) was considered a failure due to the lack of stabilization of the available microcoils in the target area at that time. A repeat procedure with different devices was planned; however, the patient was subsequently lost to follow-up. A classic retrograde approach was used in both unsuccessful intervention attempts.

During the procedure, none of the patients experienced major complications. The median fluoroscopy time was 21.6 (8–93) minutes. Immediate post-procedural angiographic imaging revealed complete occlusion in 14 patients (61%) who underwent device implantation. Echocardiographic evaluation at discharge showed no fistula-related flow in 18 patients (78%). Of the 5 patients with residual flow observed at discharge, 2 presented with moderate residual shunting, and 3 with trivial residual shunting.

### 3.4. Follow-Up

Follow-up data were available for 35 of the 42 patients, corresponding to an overall follow-up completeness of 82.5%. Seven patients had no available follow-up data and were therefore recorded with a follow-up duration of 0 months. The median follow-up duration for the entire cohort was 40 (0–270) months. Among those who underwent intervention, 3 patients were lost to follow-up, while 4 patients in the non-intervention group did not return for follow-up after the procedure. The median follow-up periods were 60 (0–270) months for the transcatheter closure group and 12 (0–126) months for the non-intervention group. Within the transcatheter intervention group, 9 patients underwent follow-up angiography, 3 underwent CTA, and 4 patients underwent both angiography and CTA. No re-interventions were performed based on angiographic findings. In the non-interventional group, which was managed without transcatheter or surgical intervention, no disease progression or symptom development was observed during the follow-up period, and no additional imaging was performed. Additional invasive or non-invasive imaging during follow-up was performed based on clinical judgment and institutional practice patterns, including assessment of coronary artery dilatation or aneurysmal changes, evaluation of coronary remodeling after fistula closure, and era-dependent follow-up strategies, rather than solely due to the presence of residual shunts.

Follow-up assessments of the 5 patients discharged with residual fistulous flow revealed that Patient 5 required surgical intervention due to persistent moderate shunting observed at the 6-month follow-up. Patients 17 and 18 exhibited complete occlusion at their 1-month follow-up appointments. Angiographic evaluation of Patient 19 demonstrated insignificant residual shunting, leading to a decision to continue monitoring without further intervention. By the end of the follow-up period, complete occlusion had been achieved in 21 of the 23 (91.3%) technically successful procedures. Furthermore, no thrombotic complications or ischemic events were observed in any of the patients during follow-up.

## 4. Discussion

In this study, we present a single-center experience with cardiac catheterization and angiographic evaluation of coronary artery fistulas in a pediatric population. In addition to the high technical success rate of transcatheter closure (92%), a substantial proportion of patients—including those managed conservatively—were asymptomatic, and no disease progression was observed during follow-up. Unlike previous echocardiography-based pediatric series, our cohort exclusively included patients who underwent diagnostic or therapeutic coronary angiography, allowing detailed morphological assessment. Furthermore, this study reflects the evolution of our institutional approach to coronary artery fistulas over a 20-year period, influenced by growing clinical experience and advances in transcatheter device technology. Nevertheless, it should be acknowledged that follow-up duration in the conservatively managed group was shorter than that of the intervention group, which may have limited the detection of late symptom development or disease progression; therefore, these findings should be interpreted with caution, and longer-term follow-up is needed to better define the natural history of conservatively managed coronary artery fistulas.

Traditionally, surgery has been the primary treatment for coronary artery fistulas. However, advances in transcatheter techniques have established percutaneous closure as a safe and effective alternative in experienced centers, even in neonates, with reported success rates ranging from 83% to over 90% [7,12,13,18,19,20]. Although some series have reported lower major complication rates with surgery, its disadvantages include longer hospital and intensive care stays and the need for cardiopulmonary bypass [21]. Consequently, surgery is currently reserved for selected cases with complex anatomy or when transcatheter closure is not feasible or complicated [7,22,23]. Furthermore, in addition to series reporting no periprocedural complications, the documented major complications have rarely caused permanent damage [12,13,19,20]. In the case series by Wang et al. involving 37 patients, tricuspid valve injury was reported in 2 cases, which occurred during AV loop formation as part of the antegrade approach [18]. In our cohort, transcatheter closure achieved a technical success rate of 92% without major complications, supporting its role as a first-line strategy in appropriately selected pediatric patients.

In the pediatric age group, many coronary artery fistulae are asymptomatic and are often detected incidentally [10,24,25]. Similar to the findings of Liberthson et al., 80.9% of patients in our series were asymptomatic at the time of diagnosis. This rate has been reported to exceed 60% in populations aged over 20 years [10]. In contrast, among patients who become symptomatic during childhood, symptoms vary according to fistula size and age, ranging from cardiac murmurs in infants to heart failure or exercise intolerance in older children [7,9,13,24]. Although rare in childhood, serious complications such as myocardial ischemia, heart failure, or aneurysm formation/rupture may develop later in life, supporting careful long-term surveillance [9,10,26,27].

Treatment decisions in our cohort were not based on a single anatomical parameter but rather on a hierarchical evaluation of multiple factors. Fistula size and drainage site were considered the primary determinants for intervention, particularly when associated with significant shunt flow or chamber enlargement. Morphology (simple versus complex) and fistula origin according to the Sakakibara classification were subsequently evaluated to assess procedural feasibility. Additional considerations included evidence of progressive chamber dilation, hemodynamic burden, and the technical suitability for safe transcatheter closure. The management of coronary artery fistulas in childhood historically involved elective intervention for asymptomatic medium-to-large fistulas, aimed at mitigating associated risks and preventing future complications. Similarly, small asymptomatic fistulas were often closed, driven by concerns regarding potential progression and the perceived increased complexity of later interventions. However, contemporary observational studies indicate that small shunts typically maintain their size, with instances of spontaneous resolution [8,15]. In keeping with contemporary observational evidence, our institution transitioned from percutaneous closure of select asymptomatic small fistulas in the early 2000 s to a predominant strategy of conservative follow-up in later years. Fistulas with minimal shunts received no interventions, and follow-up observations confirmed no procedural requirements in these patients. In our cohort, there was a general tendency toward transcatheter closure in patients with medium-to-large coronary artery fistulas, even when asymptomatic, reflecting concerns regarding potential long-term complications and the anticipated benefits of early intervention. Conservative management was reserved for a limited number of asymptomatic patients with medium-to-large fistulas in whom intervention was deferred due to complex fistula anatomy, an increased risk of occluding normal coronary branches during the procedure, or the presence of additional cardiac conditions requiring surgical management. Similarly, selected complex coronary artery fistulas were considered suitable for transcatheter closure only when a dominant and accessible drainage channel was present and device deployment could be achieved safely without compromising native coronary artery flow.

**Figure 1:**
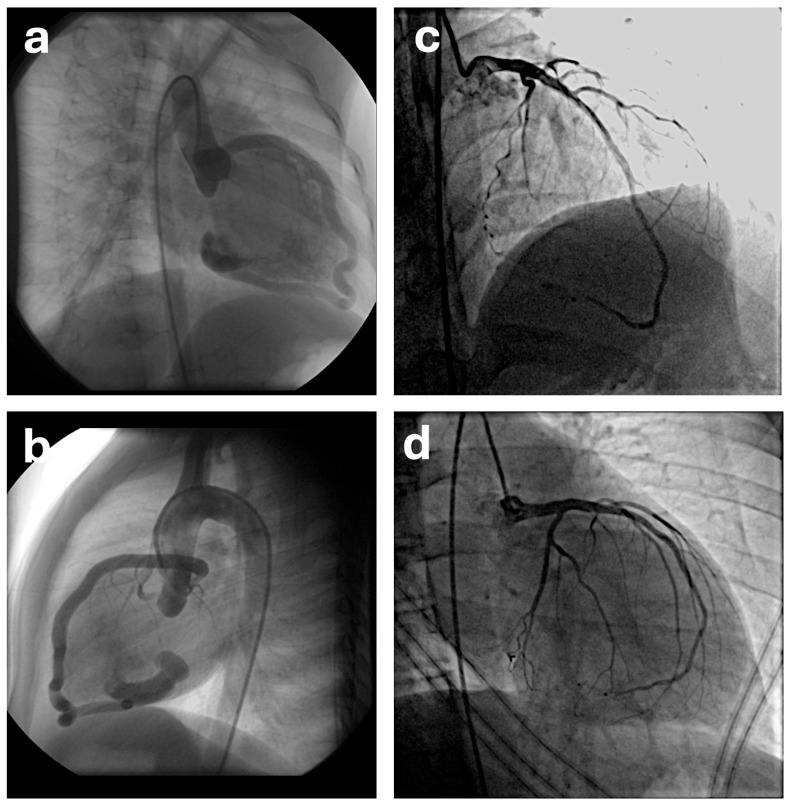
Angiographic demonstration of long-term coronary remodeling in Patient 15. (**a**,**b**) Pre-procedural selective coronary angiography showing a distal coronary artery fistula between the left coronary artery and the right ventricle. Transcatheter closure was performed using an Additional Size Amplatzer Duct Occluder. (**c**,**d**) Follow-up coronary angiography obtained at 155 months after the procedure demonstrating complete fistula occlusion and marked remodeling of the left coronary artery with restoration of normal distal coronary flow.

The conservative clinical follow-up approach applied in cases of asymptomatic small fistulas is also a viable option for medium and large-sized fistulas. However, medium and large fistulas frequently tend to progress [9,28]. In adults, advancing age introduces accumulating comorbidities that heighten procedural risks, and owing to the unpredictable nature of complications—even if rare—elective closure interventions are recommended [11]. Indications for coronary artery fistula closure in children generally include symptomatic cases with hemodynamically significant shunts (e.g., heart failure and myocardial ischemia), medium-to-large-sized fistulas, and patients with a history or risk of endocarditis. However, the timing of closure for medium-to-large asymptomatic fistulas during childhood remains controversial. Short-segment aneurysmal dilatations in proximal coronary artery-originating fistulas confer a high rupture risk, while coronary artery dilatations in distal fistulas predispose to slow flow-associated thrombotic complications with delayed closure; thus, childhood intervention during follow-up is generally recommended [9]. In addition, early closure facilitates more favorable remodeling (Figure 1) and potentially superior long-term outcomes, as supported by experienced centers’ data on successful transcatheter approaches even in neonates [13,14,19].

Over the past two decades, advances in device technology have significantly influenced transcatheter management of coronary artery fistulas. While N-butyl cyanoacrylate was used in early cases, contemporary practice favors coils, vascular plugs, and ductal occluders due to improved safety and repositionability. [13,14,18]. Contemporary device and procedural approach selection is primarily guided by the fistula’s anatomical characteristics—such as origin, drainage site, tortuosity, and size—enabling long-term successful outcomes even in small infants through the availability of diverse, low-profile options tailored to neonatal and infant anatomy. The AV loop technique facilitates stable device deployment in pediatric patients with challenging anatomy, although it may be associated with longer procedure times and rare valvular complications [14,18]. In our series, the AV loop method was applied in 6 cases without complications, whereas retrograde arterial access was favored for device implantation in the remaining patients due to its directness and lower valvular risk. These approaches exhibit variability across the literature, contingent upon anatomical features and institutional expertise, yet our hybrid strategy aligns with contemporary best practices yielding technical success rates of 83–100% [7,12,13,18,19,20].

Although earlier classical angiographic series traditionally described the right coronary artery as the most frequent origin of coronary artery fistulas [29], recent imaging-based studies increasingly demonstrate a predominance of left coronary origins, particularly the left anterior descending artery [6,15,30]. In our angiographically evaluated cohort, most CAFs originated from the left coronary artery, whereas those from the right coronary artery tended to be medium-to-large-sized and more complex. Additionally, 85% of all fistulas drained into right-sided cardiac chambers—predominantly the right ventricle and pulmonary artery—confirming right-heart drainage as the prevailing physiological endpoint. This shift in origin frequency over time can be explained by hemodynamically insignificant fistulas frequently originating from the left coronary artery and being detected more often due to advancing imaging methods. According to the Sakakibara classification, proximal (Type A) fistulas were slightly more prevalent than distal (Type B) fistulas [14], a finding of particular clinical relevance because proximal fistulas generally provide a more favorable anatomical substrate for transcatheter closure and are associated with a lower risk of distal myocardial ischemia [24]. Furthermore, nearly one-third of the cohort exhibited complex fistula morphology, underscoring the anatomical heterogeneity of CAFs and the need for individualized procedural planning. Overall, these findings are consistent with contemporary imaging series reporting increasing recognition of left coronary origins, diverse fistula morphologies and predominant right-heart drainage.

Residual shunting after transcatheter closure of coronary artery fistulas is common, especially with complex anatomy or large fistulas [9,13]. In our cohort, 22% showed residual flow at discharge (mostly trivial or mild), with most resolving spontaneously early in follow-up for a >90% complete occlusion rate among successful procedures. This aligns with prior reports small residuals often close via endothelialization and thrombosis [12,13,19]. Persistent moderate or significant shunts may require re-intervention or surgery; only one patient in our series needed surgery for ongoing moderate flow, indicating rare clinical issues with proper patient selection and devices. No thrombosis, ischemia, or device complications occurred long-term, even with early residuals. Thus, conservative monitoring suits trivial/mild shunts, with re-intervention for persistent, worsening, or symptomatic cases. Our data show that immediate angiographic occlusion is not the only success metric; long-term clinical and echocardiographic outcomes are equally vital.

This study has several limitations. It constitutes a single-center, retrospective analysis involving a small sample size, thereby limiting generalizability. Follow-up duration varied, with some patients lost to follow-up and incomplete standardized long-term imaging, which may introduce bias. Treatment decisions were non-randomized and influenced by patient characteristics and physician preferences. Nonetheless, this represents one of the largest pediatric cohorts of coronary artery fistulas evaluated by angiography with extended follow-up.

## 5. Conclusions

Transcatheter closure of coronary artery fistulas in children is safe and effective when anatomically suitable. Advancements in clinical experience and technological innovations have evolved treatment indications and device selection. Conservative follow-up is appropriate for selected asymptomatic patients with small, hemodynamically insignificant fistulas, whereas management should be individualized based on anatomical complexity, shunt severity, and long-term risks.

## Figures and Tables

**Table 1:** Characteristics of the Patients.

Variables	Patients (*n*: 42)
Male, *n* (%)	26 (61.9)
Age at catheterisation, mean ± ss (range)	7.4 ± 5.9 (3 days–18 years)
Weight at catheterisation, median (range)	23 (3.5–75)
Symptoms *, *n* (%)	
None	34 (80.9)
Shortness of breath	4 (9.5)
Palpitation	2 (4.7)
Chest pain, nonspecific	3 (7.1)
Clinical findings, *n* (%)	
Murmur	32 (76.1)
Congestive heart failure	2 (4.7)
Endocarditis, fever	1 (2.3)
ECG abnormalities, *n* (%)	
Nonspecific findings	3 (7.1)
Ischemic	0 (0)
Transthoracic echocardiography	
Systolic dysfunction, *n*(%)	0 (0)
Heart chamber dilatation, *n*(%)	8 (19)

* Symptoms were not mutually exclusive, and some patients presented with more than one symptom.

**Table 2:** Characteristics of Coronary Artery Fistulas.

Origins of CAF		Left Coronary Artery, *n* = 30 (71.4%)	Right Coronary Artery, *n* = 12 (28.6%)	Total *n* = 42 (100%)
Drainage Sites				
Right Heart Chambers *n* = 36 (85%)	Right ventricle	11	5	16 (38.1%)
	Pulmonary artery	11	2	13 (30.9%)
	Right atrium	1	3	4 (9.5%)
	Coronary sinus	1	1	2 (4.8%)
	Superior vena cava	0	1	1 (2.4%)
Left Heart Chambers *n* = 6 (15%)	Left ventricle	2	0	2 (4.8%)
	Left atrium	4	0	4 (9.5%)
Sakakibara Classification				
	A (proximal)	16	7	23 (54.7%)
	B (Distal)	14	5	19 (45.3%)
Classification according to fistula size				
	Small	10	2	12 (28.6%)
	Medium	7	5	12 (28.6%)
	Large	5	1	6 (14.2%)
	Complex	8	4	12 (28.6%)

**Table 3:** Angiographic, Procedural, and Follow-Up Characteristics of Patients Undergoing Transcatheter Closure of Coronary Artery Fistulas.

Patient No.	Year of Treatment	Age at Procedure	Weight (kg)	Sakakibara	Morphology, Size	Origin	Drainage	Closure Approach	AV Loop	Occlusion Device	Complete Occlusion		Follow-Up
											At Discharge	At Follow-Up	Duration, Months
1	2003	18 y	75	A	Basic, small	LAD	PA	R	-	N-butyl cyanoacrylate	+	+	12
2	2006	14.1 y	23	B	Complex	LAD, CX	LV	R	-	Microcoil	+	trivial	228
3	2006	10 months	11	B	Basic, medium	LAD	RV	R	-	Microcoil	+	+	29
4	2006	2.9 y	14	B	Basic, medium	LCA	RV	R	-	Microcoil	+	+	226
5	2006	3 days	3.5	A	Complex	RCA	RV	R	-	Vascular Plug	moderate	moderate	198
6	2007	1 y	8	A	Basic, medium	LAD	RV	R	-	-	failed procedure, clinical follow-up		126
7	2007	4 y	17	A	Basic, large	RCA	RA	R	-	Vascular Plug	+	+	40
8	2008	8 months	10	B	Basic, medium	LCA	RV	A	+	PFM Coil	Moderate	2. month	33
9	2008	15.3 y	56	B	Basic, large	LCA	LV	R	-	Vascular Plug	+	+	68
10	2008	3 months	5	A	Basic, small	RCA	RV	A	+	ADO II	+	+	169
11	2010	1 y	11	B	Complex	RCA	RV	R	+	Microcoil	+	+	60
12	2011	5.8 y	23	A	Complex	LCA	LA	R	-	Microcoil	Failed procedure, planned with different devices		0
13	2011	8 months	8	B	Basic, large	LCA	RV	A	+	Vascular Plug	+	NA	0
14	2012	5.7 y	26	A	Complex	LCA	LA	R	-	Microcoil	+	+	90
15	2012	2.7 y	15	B	Basic, medium	LCA	RV	R	-	ADO II AS	+	+	155
16	2012	8.7 y	26	B	Complex	LCA	LA	R	-	Microcoil	+	+	5
17	2015	6.9 y	18	A	Basic, large	LAD	RV	R	-	Vascular Plug	Trivial	1. month	47
18	2015	2.1 y	15	B	Basic, medium	RCA	RV	R	-	Vascular Plug	Trivial	1. month	84
19	2017	4.4 y	18	B	Basic, medium	RCA	RV	R	-	ADO II AS	Trivial	trivial	40
20	2017	1.9 y	10	A	Basic, large	CX	RV	A	+	Vascular Plug	+	+	15
21	2019	5.5 y	16	B	Basic, medium	CX	CS	A	+	ADO II	+	+	67
22	2019	17.6 y	64	B	Basic, medium	RCA	CS	R	-	Microcoil	+	+	30
23	2020	12.4 y	58	A	Basic, medium	RCA	RA	R	-	Microcoil	+	+	44
24	2022	5 months	6	A	Basic, medium	LCA	RA	A	+	ADO II	+	NA	0
25	2022	13.3 y	57	A	Complex	RCA	RA, VCS	R	-	Piccolo	+	+	3

Footnote: LAD, left anterior descending artery; CX, circumflex artery; LCA, left coronary artery; RCA, right coronary artery; PA, pulmonary artery; LV, left ventricle; RV, right ventricle; LA, left atrium; CS, coronary sinus; RA, right atrium; VCS, vena cava superior; R, retrograde; A, antegrade; AV, arteriovenous; ADO, Amplatzer Duct Occluder; AS, additional size; NA, not available. Patients with 0 months of follow-up represent cases without available follow-up data.

## Data Availability

The data presented in this study are available on request from the corresponding author. The data are not publicly available due to institutional restrictions.

## Abbreviations

The following abbreviations are used in this manuscript:

CAF	Coronary artery fistula
CTA	Computed tomography angiography
ECG	Electrocardiography
TTE	Transthoracic echocardiography
RCA	Right coronary artery
LCA	Left coronary artery
LAD	Left anterior descending artery
CX	Circumflex artery
CS	Coronary sinus
LA	Left atrium
RA	Right atrium
VCS	Vena cava superior
AV	Arteriovenous
R	Retrograde
A	Anterograde
NBCA	N-butyl cyanoacrylate
Qp/Qs	Pulmonary-to-systemic flow ratio
PDA	Patent ductus arteriosus
ICU	Intensive care unit
